# Public Awareness of Hearing Aids and Cochlear Implants: A Google Trends Analysis of Media Campaigns

**DOI:** 10.1002/oto2.70160

**Published:** 2025-10-16

**Authors:** Daniel R. S. Habib, Anthony E. Bishay, Alexander J. Langerman, Kareem O. Tawfik

**Affiliations:** ^1^ Vanderbilt University School of Medicine Nashville Tennessee USA; ^2^ Department of Otolaryngology–Head and Neck Surgery Vanderbilt University Medical Center Nashville Tennessee USA; ^3^ Department of Radiology and Radiological Sciences Vanderbilt University Medical Center Nashville Tennessee USA; ^4^ Center for Biomedical Ethics and Society Vanderbilt University Medical Center Nashville Tennessee USA

**Keywords:** cochlear implant, Google Trends, hearing aids, hearing loss, internet

## Abstract

**Objective:**

While one‐fifth of eligible candidates use hearing aids (HAs), a smaller proportion of eligible candidates receive a cochlear implant (CI), partly due to cost and knowledge gaps. This study aims to quantify internet relative search volumes (RSVs) as a general awareness proxy for HAs and CIs around relevant events.

**Study Design:**

Retrospective infodemiologic study.

**Setting:**

Google searches from 2004 to 2024.

**Methods:**

Using Google Trends, we performed Welch's *t*‐tests to compare average HA and CI RSVs (search volume relative to comparator search volume from 0%‐100%) during event and non‐event periods.

**Results:**

From 2004 to 2024, HA RSV increased from 37% to 100% while CI RSV remained below 13%. Some federal announcements such as hybrid CI approval (Mar 2014; *P* < .045) and a new over‐the‐counter HA category (Oct 2021; *P* < .001) coincided with significantly increased CI and HA RSVs, respectively, while others like the Nov 2024 federal announcement stating when all cell phones must have wireless connectivity with HA (*P* = .099) and CI (*P* = .777) did not. Similarly, some public awareness campaigns like “Hearing 20/20” (*P* < .001 for HA and CI) and Feb 2024 International CI Day (*P* < .001) coincided with significantly increased RSVs, while others like the Feb 2009 (*P* = .093) and Feb 2023 International CI Days (*P* = .327) did not.

**Conclusion:**

This study highlights that, unlike increasing search activity around HAs, CIs have not exhibited the same substantial changes in search volume, aside from brief spikes around certain campaigns. These findings underscore the need for more effective and sustained public outreach strategies to improve hearing device awareness.

Over 1 in 5 children and adults in the United States experience hearing loss.^1^, ^2^ A substantial proportion of Americans could benefit from assistive listening devices (ALDs), including hearing aids (HAs) and cochlear implants (CIs), with varying rates of utilization.^1^, ^3^, ^4^ Around 21% of the 40 million Americans who would benefit from HAs use them.^3^ In contrast, estimates of Americans who would benefit from CIs range from 8 million using expanded criteria to 1.3 million using traditional audiometric criteria, with 2.1% to 12.7% receiving CIs, respectively.^4^ ALD underutilization in the United States is multifactorial. Some factors are cost, personal denial of needing ALDs (especially in individuals with milder hearing loss), low device satisfaction, social stigma, lack of support, and sociopolitical issues surrounding deafness.^5^, ^6^ Some unique reasons for low CI versus HA utilization include fear of surgery, insurance coverage or higher out‐of‐pocket cost, and knowledge gaps among the general public and referring providers about surgical candidacy and outcomes.^5^, ^7^

Google Trends, a tool for measuring internet search activity, is commonly used to provide insights on wide‐ranging health‐related patterns.^8^ To better understand public interest and awareness, researchers have increasingly used internet search activity like Google Trends’ relative search volume (RSV) as a proxy for public engagement with health topics. RSV reflects the proportion of searches for a given term relative to all Google searches in a specified time and location, scaled from 0 to 100. Examples in the literature include surveillance or correlating specific events with internet activity trends for health topics such as abortion,^9^ smoking,^10^, ^11^ infection outbreaks,^12^ cancer awareness campaigns,^13^ LASIK surgery public interest,^14^ mental health search seasonality,^15^ anesthesia interest,^16^ pharmaceutical utilization,^17^ otolaryngology,^18^ and many more.^8^ Previous research has also leveraged internet search activity to assess CI public awareness following significant events. One study examined CI‐related search activity during a single month (October 2022), highlighting an increase in HA searches following the US Food and Drug Administration (FDA)'s approval of over‐the‐counter (OTC) hearing aids.^19^ Additionally, two studies showed an increase in U.S. CI internet searches around International Cochlear Implant Day between 2016 and 2021.^20^, ^21^

Despite these prior studies, no work has studied CI trends associated with other public awareness events or HA longitudinal trends outside of one year. Additionally, the impact of federal announcements has not been analyzed. This study aims to assess the relative difference in general awareness between HAs and CIs as well as changes in internet searches for HAs and CIs activity associated with hearing loss‐related public awareness campaigns and federal announcements.

## Methods

Briefly, we summarized key characteristics of HAs and CIs, including indications, intended effects, side effects, and device lifespan. Google Trends was leveraged to compare patterns in internet search behavior as a proxy for general awareness, particularly around significant events. Lastly, we extracted Medicare data to calculate rough estimates of submitted charges and reimbursements. All analyses were performed using IBM SPSS Statistics 27 (IBM), and figures were created using Prism (GraphPad). Adherence to Strengthening the Reporting of Observational Studies in Epidemiology (STROBE) guidelines was ensured.

### Google Trends

Google Trends is a publicly available tool for collecting monthly search volume data for specific terms over a specific time frame and region.^22^ Results are given as data ranging from 0 to 100, normalized to search volume peak within the specified period. The data represent RSVs for each search term. Thus, values are not absolute search counts but rather relative measures over time specific to the comparison of interest, reported as percentages. RSVs must be taken within the context of the year in which they were extracted.

Google Trends was used to retrieve monthly RSV data for the exact terms “hearing aids” and “cochlear implant.” Data encompass searches conducted within the United States from January 1, 2004, to December 15, 2024. Of note, Google Trends data were not available before 2004. Results were standardized to the month with the highest search volume for “hearing aids” (December 2024), and the monthly RSV trends for each search term were graphed to facilitate comparison.

To study event RSVs more closely, mean monthly RSV values and percentage changes were determined for specific periods of interest––that is, those preceding or associated with significant events based on RSV data of that year. Events were initially drawn from those examined in prior studies,^19^, ^20^, ^21^ and this list was supplemented with relevant examples from targeted internet searches. Google Trends offers week‐level granularity only when extracting data from an individual year. Rather than only looking at the overall trend from 2004 to 2024, we also extracted year‐level RSV data. Therefore, for each event year, RSVs were scaled relative to the week with the highest search volume within that year, whereas RSVs for the overall 2004–2024 period were scaled relative to the month with the highest search volume across the entire timeframe. RSVs during event and non‐event time periods were compared using a validated approach.^21^ Event time period was defined as two weeks before and after. Nonevent period was defined as all weeks of the same calendar year excluding the four‐week event period. To ensure comparisons were independent, event windows were non‐overlapping and analyzed separately. Average and standard deviation (SD) of weekly RSV values during event and non‐event periods were calculated and compared using Welch's *t*‐tests, consistent with prior Google Trends analyses.^23^ This approach accounts for unequal variances and allows for formal testing of whether an observed change exceeds expected variability, rather than relying solely on percent change.

## Results

### Key Characteristics and Timeline

**Figure 1:**
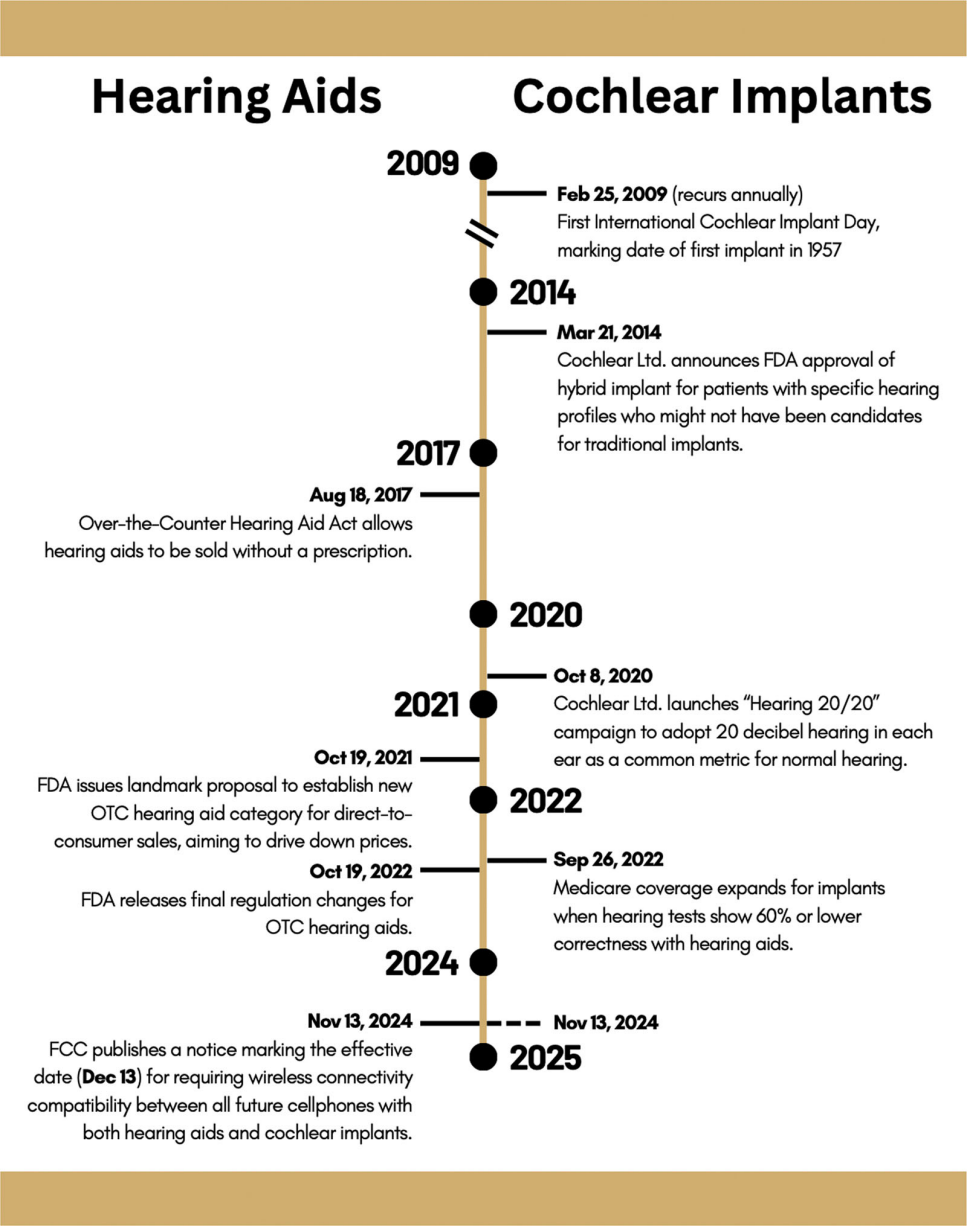
Timeline of pertinent significant events for hearing aids and cochlear implants.

Prescription HAs have helped amplify sound to improve speech understanding and environmental awareness for decades. However, it was enactment of the Over‐the‐Counter Hearing Aid Act on August 18, 2017 that allowed HAs to be widely accessible in the United States (Figure 1).^24^ After expansion of HA accessibility, the FDA issued a landmark proposal in October 2021 to establish a new OTC HA category that allowed HAs to be sold direct‐to‐consumer to increase competition and drive down prices.^25^ The FDA established the proposed regulation changes exactly a year later.^24^ Recently, the Federal Communications Commission (FCC) published a notice marking the effective date (December 13, 2024) for requiring wireless connectivity compatibility between all future cellphones with HAs and CIs after a transition period.^26^ In sum, there has recently been substantial expansion of HA accessibility and compatibility.

CIs often serve as a step up from HAs but are associated with unique characteristics and significant events (Table 1). While HAs can be used for mild to severe sensorineural or conductive hearing loss, CIs are indicated only for sensorineural hearing loss. The First International Cochlear Implant Day 2009 occurred on February 25, 2009, marking the date of the first cochlear implantation in 1957.^27^ On March 21, 2014, Cochlear Ltd. announced FDA approval of their hybrid implant for specific hearing profiles, expanding the patient population that can be helped.^28^ On October 8, 2020, Cochlear Ltd. launched their “Hearing 20/20” campaign, calling for adoption of 20 decibel hearing in each ear as a common metric for normal hearing.^29^ September 26, 2022 was a landmark day for CI accessibility as Medicare coverage expanded to include patients who experienced limited benefit from HAs (ie, 60% or worse sentence recognition while using HAs).^30^

**Table 1:** Key Characteristics of Hearing Aids and Cochlear Implants

Characteristic	Hearing aids	Cochlear implant
Indications	Mild to severe sensorineural hearing loss or conductive hearing loss, typically diagnosed with an audiogram	Severe to profound bilateral or unilateral sensorineural hearing loss; limited benefit from hearing aids (≤60% sentence recognition for Medicare coverage)
Intended effects	Amplifies sound to improve speech understanding and environmental awareness	Converts sound into electrical signals that directly stimulate the auditory nerve, restoring access to speech and environmental sounds in cases of severe hearing loss
Side effects	Feedback (whistling sound), discomfort/irritation in ear canal, limited benefit in severe hearing loss	Dizziness/vertigo, surgical risks (eg, infection), altered taste or tingling sensation due to nerve proximity
Device lifespan	3‐7 years	5‐10 years for external processor
		10‐25 years for internal implant

### Overall Relative Search Volume

**Figure 2:**
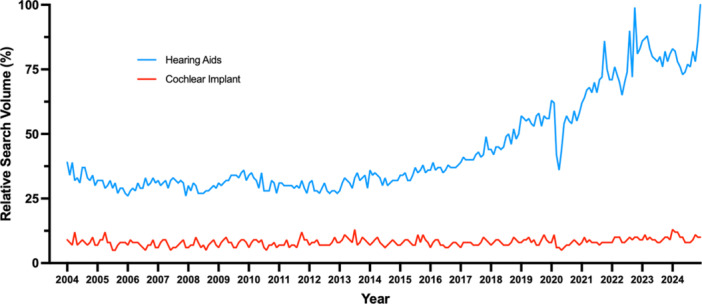
US Internet Search volumes for hearing aids and cochlear implant from January 2004 to December 2024.

Between January 2004 and December 2016, the RSV for “hearing aids” remained stable (Figure 2), ranging between 26 and 39. From December 2016 (RSV = 39%) to October 2021 (RSV = 86%), the RSV steadily increased by 120.5%, reaching its peak in October 2021 during the second Audiology Awareness Month of Cochlear Ltd.‘s “Hearing 20/20” campaign. Peaks were observed in October 2022 (RSV = 99%) with the FDA's release of final OTC HA regulations and December 2024 (RSV = 100%) when the FCC's requirement for wireless connectivity in hearing devices took effect. On the other hand, the RSV for “cochlear implant” remained consistently low, never exceeding 13%.

### Relative Search Volume Changes by Event Type

#### Public Awareness Campaigns

International Cochlear Implant Day coincided with variable search activity depending on the year (Table 2). In February 2009, CI searches increased slightly compared to the yearly average (21.7 ± 1.2% vs 20.3 ± 4.5%, *P* = .093), while HA searches significantly declined (79.0 ± 1.9% vs 83.5 ± 6.5%, *P* < .001). In October 2020, the “Hearing 20/20” Campaign coincided with significantly increased searches for both devices, with CI searches rising to 14.3 ± 0.8% from 12.2 ± 9.1% (*P* < .001) and HA searches increasing to 89.8 ± 3.8% from 82.8 ± 11.8% (*P* < .001). Around International Cochlear Implant Day in February 2022 over a decade after the first, CI searches remained stable (6.0 ± 0.9% vs 5.6 ± 0.8%, *P* = .360), while HA searches showed a slight but significant decline (41.3 ± 1.5% vs 44.3 ± 9.7%, *P* = .045). The following year (February 2023), CI searches remained unchanged (10.6 ± 0.9% vs 10.1 ± 1.3%, *P* = .327), whereas HA searches significantly increased (88.0 ± 2.1% vs 80.8 ± 7.0%, *P* < .001). By February 2024, CI searches rose significantly (11.8 ± 0.4% vs 9.7 ± 2.6%, *P* < .001), while HA searches showed no significant change (73.0 ± 3.1% vs 73.8 ± 7.5%, *P* = .633).

**Table 2:** Cochlear Implant and Hearing Aid Relative Search Volumes Around Significant Events

Event	HA event RSV (%), average (SD)	HA year RSV (%), average (SD)	Statistic	CI event RSV (%), average (SD)	CI year RSV (%), average (SD)	Statistic
First International Cochlear Implant Day (Feb 2009)	79.0 (1.9)	83.5 (6.5)	*t* = −3.83; *P* < .001***	21.7 (1.2)	20.3 (4.5)	*t* = 1.74; *P* = .093
FDA Approval for Hybrid Implant (Mar 2014)	94.0 (3.8)	90.9 (28.6)	*t *= 1.68; *P* = .144	27.0 (3.9)	22.1 (3.9)	*t* = 2.66; *P* = .045*
Over‐the‐Counter Hearing Aid Act (Aug 2017)	80.0 (4.4)	81.4 (5.1)	*t* = −0.66; *P* = .535	13.8 (1.5)	15.1 (2.4)	*t* = −1.80; *P* = .121
“Hearing 20/20” Campaign (Oct 2020)	89.8 (3.8)	82.8 (11.8)	*t* = 3.13; *P* < .001***	14.3 (0.8)	12.2 (9.1)	*t* = 4.07; *P* < .001***
FDA Establishes New OTC Hearing Aid Category (Oct 2021)	92.3 (5.8)	78.4 (7.0)	*t* = 5.46; *P* < .001***	9.8 (0.8)	9.5 (1.5)	*t* = 0.78; *P* = .456
International Cochlear Implant Day (Feb 2022)	41.3 (1.5)	44.3 (9.7)	*t* = −2.05; *P* = .045*	6.0 (0.9)	5.6 (0.8)	*t* = 0.99; *P* = .360
Medicare Expands Coverage for CIs (Sep 2022)	43.0 (2.5)	44.3 (9.7)	*t* = −0.77; *P* = .453	6.0 (0.7)	5.6 (0.8)	*t* = 1.12; *P* = .310
FDA Releases Final OTC Hearing Aid Regulations (Oct 2022)	56.5 (21.8)	44.3 (9.7)	*t* = 1.35; *P* = .235	6.5 (0.5)	5.6 (0.8)	*t* = 3.53; *P* = .008**
International Cochlear Implant Day (Feb 2023)	88.0 (2.1)	80.8 (7.0)	*t* = 5.37; *P* <.001***	10.6 (0.9)	10.1 (1.3)	*t* = 1.07; *P* = .327
International Cochlear Implant Day (Feb 2024)	73.0 (3.1)	73.8 (7.5)	*t* = −0.49; *P* = .633	11.8 (0.4)	9.7 (2.6)	*t* = 5.25; *P* < .001***
FCC Publishes Date for Wireless Connectivity Requirement (Nov 2024)	81.7 (9.5)	73.8 (7.5)	*t* = 1.90; *P* = .099	9.8 (1.0)	9.7 (2.6)	*t* = 0.29; *P* = .777

RSVs in this table cannot be compared across years as the max RSV used to calculate the RSVs throughout the rest of the year is different for each year. Significance is denoted as *P < .05, **P < .01, and ***P < .001.

Abbreviations: CI, cochlear implant; FCC, Federal Communications Commission; FDA, US Food and Drug Administration; HA, hearing aid; OTC, over‐the‐counter; RSV, relative search volume; SD, standard deviation.

#### Federal Announcements

Several regulatory actions by the FDA and other agencies coincided with changes in search trends. Following FDA approval for the hybrid CI (March 2014), CI searches significantly increased (27.0 ± 3.9% vs 22.1 ± 3.9%, *P* = .045), while HA searches remained unchanged (94.0 ± 3.8% vs 90.9 ± 28.6%, *P* = .144). The Over‐the‐Counter Hearing Aid Act announcement (August 2017) did not coincide with a significant effect on CI searches (13.8 ± 1.5% vs 15.1 ± 2.4%, *P* = .121) or HA searches (80.0 ± 4.4% vs 81.4 ± 5.1%, *P* = .535). When the FDA established a new OTC HA category (October 2021), CI searches remained stable (9.8 ± 0.8% vs 9.5 ± 1.5%, *P* = .456), while HA searches significantly increased (92.3 ± 5.8% vs 78.4 ± 7.0%, *P* < .001). Search trends remained stable following policy changes related to insurance coverage. In September 2022, Medicare Coverage Expansion for CIs did not coincide with significant differences in CI searches (6.0 ± 0.7% vs 5.6 ± 0.8%, *P* = .310) or HA searches (43.0 ± 2.5% vs 44.3 ± 9.7%, *P* = .453). Counterintuitively, the FDA's release of final OTC HA regulations (October 2022) coincided with a significant increase in CI searches (6.5 ± 0.6% vs 5.6 ± 0.8%, *P* < .001), while HA searches remained unchanged (56.5 ± 21.8% vs 44.3 ± 9.7%, *P* = .235). In November 2024, the FCC's announcement of when cell phones would be required to have wireless connectivity capabilities with ALDs did not coincide with a significant change for CI searches (9.8 ± 1.0% vs 9.7 ± 2.6%, *P* = .777) but did coincide with a non‐significant trend toward increased HA searches (81.7 ± 9.5% vs 73.8 ± 7.5%, *P* = .099).

## Discussion

The current analysis reveals several key patterns in public interest surrounding ALDs from 2004 to 2024. HA‐related searches showed a dramatic 120.5% increase from December 2016 to October 2021, with peak interest coinciding with major regulatory events, particularly the FDA's OTC HA regulations (October 2022) and the FCC's wireless connectivity requirement (December 2024). In contrast, CI searches maintained consistently low levels throughout the study period, with surprisingly minimal change occurring around the time of significant policy changes relating to Medicare criteria for implantation.

Google Trends has emerged as a valuable tool in healthcare research, particularly for understanding public awareness and response to significant events.^8^ For hearing technology specifically, this tool provides unique insights into how policy changes and awareness campaigns might coincide with public interest and information‐seeking behavior. While dedicated events like International Cochlear Implant Day sometimes coincided with periodic spikes in interest, sustained changes in search behavior may be limited, raising questions about long‐term effectiveness of current awareness campaigns. The disparity in search patterns between HAs and CIs likely reflects their different target populations and accessibility levels. HAs, serving a broader population, consistently generate higher search volumes, driven by their wider applicability and recent regulatory changes that have increased both accessibility and mainstream attention to hearing healthcare.

In contrast, Medicare expansion for CIs in September 2022 marked a significant policy shift but did not coincide with any significant change in search behavior. Given the dramatic impact of this policy shift in expanding the pool of potential CI‐eligible Medicare patients, this observation was unexpected and may reflect a concerning gap between policy implementation and public awareness of new opportunities for hearing healthcare. This communication gap is particularly relevant in the context of CIs, where adoption is influenced by an interplay of factors including regional disparities in utilization, significant differences in qualification criteria, and substantial access barriers. Importantly, limited search activity surrounding expanded coverage may reflect both the narrower eligibility for CIs compared to HAs and a broader disconnect between policy changes and public awareness. This suggests a need for more targeted and timely communication strategies to ensure eligible patients are informed about available resources.

Cost considerations remain crucial in helping patients reconnect with the world around them. According to Medicare^31^ charge and reimbursement data for Current Procedural Terminology (CPT) codes associated with HA and CI services as well as cost estimates from prior studies adjusted for inflation (Supplemental Table S1, available online),^3^, ^4^, ^32^, ^33^ one prescription HA costs approximately $2400 while a CI costs about $34,200, with implantation surgery averaging over $10,000 in submitted charges (Supplemental Table S2, available online). Average CI reimbursement rates vary substantially both nationally and internationally.^34^, ^35^ While OTC HAs have expanded accessibility, CI surgery remains a more significant intervention, requiring stricter medical qualifications and higher barriers to entry, particularly for certain demographics.

Limited insurance coverage for ALDs poses a substantial barrier, with the Veterans Health Administration being a notable exception in providing comprehensive coverage, leading to higher CI utilization among veterans.^3^, ^36^, ^37^ Regarding HAs, the new OTC category has introduced more affordable options ($200‐$1000), contrasting sharply with prescription HAs ($1000‐$6000).^38^ For children who receive hearing‐assistive technology or not, tailored education costs must also be considered, with deaf education programs costing from $20,000 to over $100,000 per child annually.^39^, ^40^, ^41^ These cost differentials, combined with varied insurance coverage, create a complex landscape of accessibility that likely influences both search patterns and ultimate device adoption.

Although cost is a major factor affecting accessibility, demographic factors significantly influence hearing loss patterns, with age being the strongest predictor among adults.^42^ The gender disparity is notable, with men, particularly non‐Hispanic white men, experiencing nearly twice the likelihood of hearing loss.^42^ These demographic patterns, combined with cost barriers and varying insurance coverage discussed above, likely contribute to observed disparities in ALD adoption. Importantly, while these demographic trends cannot be captured directly through Google Trends, they provide important context for understanding population‐level disparities in hearing device utilization.

While the current analysis shows growing public interest in hearing healthcare solutions, particularly around major policy changes, the persistent gap between search patterns and policy initiatives suggests that significant barriers to access and adoption remain. Future initiatives should focus on sustained awareness campaigns that specifically target underserved demographic groups, address cost barriers, and ensure equitable access to bridge the gap between those who need hearing assistance devices and those who ultimately obtain them. Successful health awareness efforts in other fields may offer useful models—for instance, the “Get Yourself Tested” (GYT) campaign for sexually transmitted infections (STIs) and the “Tips From Former Smokers” campaign have both demonstrated how strategic use of media, community outreach, and partnerships with trusted institutions can drive engagement and behavior change.^43^, ^44^ Adapting similar strategies—such as culturally tailored messaging, collaborations with primary care providers, and targeted social media outreach—may help improve public awareness and adoption of hearing healthcare solutions.

### Limitations

Some limitations must be considered when using Google Trends data as a research tool. Causal inferences cannot be drawn from these data. Google Trends data do not directly reflect public awareness of hearing healthcare solutions. Instead, these data serve as a proxy for public awareness via relative search traffic, which might be better categorized as a measurement of public interest. Unexpected fluctuations in search volume may reflect unrelated variation or random noise rather than meaningful changes in public interest, highlighting the importance of interpreting these trends with caution. Although some RSV changes reached statistical significance, these findings should be interpreted as markers of relative public interest, not clinical outcomes or behavioral change. Moreover, significance of an observed change is influenced by variance within the dataset; thus, seemingly small changes may be statistically significant in low‐variance settings, while larger changes may not reach significance in high‐variance datasets. To best account for this, we used statistical best practices for Welch's *t*‐tests,^45^ using a more stringent significance threshold than that used in other work.^23^ Moreover, our analysis focused on the search terms “hearing aids” and “cochlear implant” due to their clarity and comparability. However, the public may use various alternative terms such as “hearing devices,” “hearing assistance,” or specific brand names that may also reflect interest in these technologies but were not captured in our search strategy. As a result, our findings may underrepresent the full spectrum of public engagement with hearing healthcare solutions. Future work could expand upon this approach using composite search terms, topic‐based tools, or natural language processing to better account for variation in how individuals seek hearing‐related information online. Additional limitations include inability to distinguish between searches conducted by healthcare professionals, researchers, patients, or the general public as well as potential geographic variations in search behavior that might not be fully captured in our analysis. Moreover, HAs and CIs represent fundamentally different treatment paradigms, which may not be directly comparable. Although OTC HAs provide a more accessible and less invasive option for hearing assistance, CIs represent a surgical intervention for more severe and different types of hearing loss. Hence, comparing public interest in these two options may have inherent limitations due to the different nature of the interventions and their target populations. Despite these limitations, Google Trends remains a powerful tool for examining public engagement with healthcare topics.^8^, ^10^, ^11^, ^18^, ^19^, ^20^, ^21^, ^46^, ^47^, ^48^

## Conclusion

Our analysis highlights differences in internet search volume between HAs and CIs over the past two decades, as reflected in Google Trends data. While HAs have seen significant increases in search activity, particularly following regulatory changes like the FDA's approval of OTC devices, CIs have not exhibited the same substantial changes in search volume, even during periods of policy expansion such as Medicare coverage extension. These findings underscore persistent barriers to CI awareness and adoption, including cost, surgical considerations, and knowledge gaps among patients and providers. Addressing these barriers requires targeted efforts to enhance public education, streamline communication about policy changes, and reduce economic and logistical hurdles. Bridging these gaps is essential for optimizing access to hearing healthcare and improving quality of life for individuals with hearing loss.

## Author Contributions

**Daniel R. S. Habib**, design, conduct, analysis, writing original draft, reviewing final draft; **Anthony E. Bishay**, design, conduct, analysis, writing original draft, reviewing final draft; **Alexander J. Langerman**, design, reviewing final draft; **Kareem O. Tawfik**, design, reviewing final draft.

## Disclosures

### Competing interests

None.

### Funding source

None.

## Supporting information

Supporting information.

## Data Availability

Data are publicly available from Google Trends.
