# Supplementary material for: Public Awareness of Hearing Aids and Cochlear Implants: A Google Trends Analysis of Media Campaigns
Source: OTO Open. 2025 Oct 16;9(4):e70160. doi: 10.1002/oto2.70160 (PMC12529450; doi:10.1002/oto2.70160)
Supplement: Supplementary file 1 — Supporting information. [file OTO2-9-e70160-s001.docx]

**Supplemental Table 1**. Relevant Current Procedural Terminology (CPT) codes Associated with Hearing Aids and Cochlear Implants

| **CPT Code** | **Description** |
| --- | --- |
| 92626 | Evaluation of auditory rehabilitation to instruct the use of residual hearing provided by an implant or hearing aid related to hearing loss |
| 69930 | Cochlear implant device implantation, with or without mastoidectomy |
| 92603 | Diagnostic analysis of cochlear implant, age 7 years or older, initial programming |

**Supplemental Table 2**. Costs of a Prescription Hearing Aid versus Cochlear Implant

|  | Prescription Hearing Aid | | Cochlear Implant | |
| --- | --- | --- | --- | --- |
| **Component of Care** | **Submitted Cost (SD)** | **Reimbursement (SD)** | **Submitted Cost (SD)** | **Reimbursement (SD)** |
| Evaluation^*^ | $234.00 ($75.39) | $61.70 ($5.93) | $234.00 ($75.39) | $61.70 ($5.93) |
| Device^†^ | $2,400 | $0 | $34,200 | - |
| Implantation Surgery^*^ | - | - | $10,431.03 ($7,985.45) | $2,800.55 ($2,737.68) |
| Programming^*^ | - | - | $366.80 ($94.81) | $114.06 ($10.94) |

^*^ Centers for Medicare & Medicaid Services (CMS)^1^

^†^ Device costs for one prescription hearing aid from a prior work’s calculation adjusting for inflation^2–4^ and device cost for one cochlear implant device from a prior work’s calculation adjusting for inflation.^4,5^ Medicare does not reimburse for the device cost of hearing aids,^2,6^ and the CMS dataset does not include Medicare reimbursement data for cochlear implant device cost.

SD: standard deviation

**References**

1. Centers for Medicare & Medicaid Services. Medicare Physician & Other Practitioners by Geography and Service. Published 2024. https://data.cms.gov/provider-summary-by-type-of-service/medicare-physician-other-practitioners/medicare-physician-other-practitioners-by-geography-and-service

2. Nassiri AM, Ricketts TA, Carlson ML. Current Estimate of Hearing Aid Utilization in the United States. *Otol Neurotol Open*. 2021;1(1):e001. doi:10.1097/ONO.0000000000000001

3. Borre ED, Dubno JR, Myers ER, et al. Model-Projected Cost-Effectiveness of Adult Hearing Screening in the USA. *J Gen Intern Med*. 2023;38(4):978-985. doi:10.1007/s11606-022-07735-7

4. U.S. Bureau of Labor Statistics. Consumer Price Index. Published 2024. https://www.bls.gov/cpi/

5. Nassiri AM, Sorkin DL, Carlson ML. Current Estimates of Cochlear Implant Utilization in the United States. *Otol Neurotol*. 2022;43(5):e558-e562. doi:10.1097/MAO.0000000000003513

6. Warren E, Grassley C. Over-the-Counter Hearing Aids. *JAMA Intern Med*. 2017;177(5):609. doi:10.1001/jamainternmed.2017.0464
